# Identification of novel drought-tolerant-associated SNPs in common bean (*Phaseolus vulgaris*)

**DOI:** 10.3389/fpls.2015.00546

**Published:** 2015-07-21

**Authors:** Emiliano Villordo-Pineda, Mario M. González-Chavira, Patricia Giraldo-Carbajo, Jorge A. Acosta-Gallegos, Juan Caballero-Pérez

**Affiliations:** ^1^Campo Experimental Bajío-Instituto Nacional de Investigaciones Forestales, Agrícolas y PecuariasCelaya, México; ^2^Unidad de Genética, Departamento de Biotecnología-Biología Vegetal, Escuela Técnica Superior de Ingenieros Agrónomos, Universidad Politécnica de MadridMadrid, Spain; ^3^Facultad de Química, Universidad Autónoma de QuerétaroQuerétaro, Mexico

**Keywords:** bean, stress, drought, SNP, biomarker, genotyping

## Abstract

Common bean (*Phaseolus vulgaris* L.) is a leguminous in high demand for human nutrition and a very important agricultural product. Production of common bean is constrained by environmental stresses such as drought. Although conventional plant selection has been used to increase production yield and stress tolerance, drought tolerance selection based on phenotype is complicated by associated physiological, anatomical, cellular, biochemical, and molecular changes. These changes are modulated by differential gene expression. A common method to identify genes associated with phenotypes of interest is the characterization of Single Nucleotide Polymorphims (SNPs) to link them to specific functions. In this work, we selected two drought-tolerant parental lines from Mesoamerica, Pinto Villa, and Pinto Saltillo. The parental lines were used to generate a population of 282 families (F_3:5_) and characterized by 169 SNPs. We associated the segregation of the molecular markers in our population with phenotypes including flowering time, physiological maturity, reproductive period, plant, seed and total biomass, reuse index, seed yield, weight of 100 seeds, and harvest index in three cultivation cycles. We observed 83 SNPs with significant association (*p* < 0.0003 after Bonferroni correction) with our quantified phenotypes. Phenotypes most associated were days to flowering and seed biomass with 58 and 44 associated SNPs, respectively. Thirty-seven out of the 83 SNPs were annotated to a gene with a potential function related to drought tolerance or relevant molecular/biochemical functions. Some SNPs such as SNP28 and SNP128 are related to starch biosynthesis, a common osmotic protector; and SNP18 is related to proline biosynthesis, another well-known osmotic protector.

## Introduction

Common bean (*Phaseolus vulgaris* L.) is the most important leguminous crop species for human nutrition because it is a natural source of essential nutrients and proteins in the diet of ~500 million people in Latin America and Africa (Broughton et al., 2003). Common bean originated in the Americas and diverged into the Mesoamerican and Andean genetic pools before domestication (Gepts, 1998; Mamidi et al., 2011; Bitocchi et al., 2013; Schmutz et al., 2014). The Mesoamerican gene pool includes the Mesoamerican, Durango, Jalisco, and Guatemala races (Singh et al., 1991; Díaz and Blair, 2006; Blair et al., 2009; Kwak and Gepts, 2009). The Mesoamerican and Durango races are considered to be a rich genetic source for drought stress resistance (Terán and Singh, 2002b; Singh, 2007).

Drought is the most important abiotic stress limiting cultivar productivity. Drought negatively impacts dry-bean cultivars depending on intensity, type and duration of the stress (Terán and Singh, 2002a,b; Muñoz-Perea et al., 2006). Sixty percent of the worldwide dry-bean production is affected by terminal or intermittent drought (Beebe et al., 2008) and it is the second most important factor in yield reduction after plant diseases (Thung and Rao, 1999; Rao, 2001). We can expect than drought will be increasing in number of events and duration in the principal agriculture regions because of global warming. This will affect negatively the production and therefore food availability (McClean et al., 2011). In particular, tropical regions, where poverty and starvation are important problems, will be more affected (Cavalieri et al., 2011). In Latin America is estimated that drought conditions can reduce seed production to 73%, or lost of production. Even worse, drought effects are increased by other biotic or abiotic factors (Polanía et al., 2012).

Drought resistance and adaptation includes several mechanisms to allow plants survive during dry periods. In general, drought resistance mechanisms can include drought escape; drought avoidance; and drought tolerance (Beebe et al., 2013). Drought escape allows plants to accelerate their cell cycle with an early flowering and maturity, and rapidly relocates metabolites to seed production (Beebe et al., 2013) and away from leaves and shoot tissues (Blum, 2005; Nakayama et al., 2007). Drought avoidance is the capability to keep a high tissue water potential through increased rooting depth, hydraulic conductance reduction, radiation absorption reduction in leaves, water-loss area reduction reduced absorption of radiation by leaf movement, and reduced evaporation surface (leaf area) (Beebe et al., 2013). Drought tolerance is the capability in plants to resist the stress by adjusting cell osmosis, cell plasticity, and cell size (Beebe et al., 2013).

Given common bean importance, the search for genetic markers to increase the efficiency of plant selection is essential. New large-scale modern genotyping technologies such as Single Nucleotide Polymorphism (SNP) arrays or Next Generation Sequencing (NGS) can be correlated with phenotypic data in germplasm collections or other useful populations (Beebe et al., 2013). Those technologies allow us the identification of key loci associated with a stress response or a particular phenotype (i.e., higher product biomass, fast development, etc.) (McClean et al., 2011).

SNPs are abundant and well distributed across the genome and can be used for genotyping with high specificity, reproducibility, and performance (Rafalski, 2002; Hyten et al., 2010; Yan et al., 2010). SNPs can be used in breeding for linkage maps construction, genetic diversity analysis, or marker-phenotype association studies for marker-assisted selection (MAS) (Cortés et al., 2011). More than 30 methods for SNP detection has been developed and applied in different species (Gupta et al., 2008). SNP detection in common bean has been applied using Single Base Extension (SBE) (Gaitán-Solís et al., 2008), Expressed Sequence Tags (EST) (Galeano et al., 2012), NGS (Hyten et al., 2010) and Kompetitive allele-specific PCR (KASP) (Cortés et al., 2011; Goretti et al., 2014). SNP information from common bean has been used to build linkage maps and synteny analysis previously (Galeano et al., 2009a,b, 2012; Shi et al., 2011; Yuste-Lisbona et al., 2012). SNPs have been used for the identification of Quantitative Trait Loci (QTL) associated with drought stress in a F_5:7_ population derived from a cross between a Mesoamerican and an Andean bean genotype (Mukeshimana et al., 2014). Cortés et al. (2011) used SNP validation from the EST reported in Galeano et al. (2009a,b) and candidate gene validation to drought adaptation in a set group of bean genotypes analyzed previously by Simple Sequence Repeat (SSR) methods in Blair et al. (2006).

Loci for complex traits have been detected in genome wide association studies (GWAS), and from those, only a small fraction of genetic variants are clearly responsible for the phenotype. Modern statistical methods allow us to identify which variants have a real effects on the phenotype providing associated probabilities (Hormozdiari et al., 2014).

We employed an simplified method derived from GWAS, the Single-Marker Analysis (SMA). This method reports the genotype-phenotype associations in a small population. Our populaton is derived from two drought-resistant lines: Pinto Villa (PV) and Pinto Saltillo (PS). PV and PS are members of the Durango race, and both have been extensively studied because they are drought-resistant in semi-desert areas in Mexico. Both lines were derived independently: PV is derivate from II-25D-M-34 = II-925-M-29-1 x ('Canario 101′ × Mex-4-2) and PS is derivate from MX 8738 = 'Hidalgo 77′/4/'MAM 30′/3/'Michoacán 91-A′/'BAT 76′//'BAT 93′/'Ecuador 299′ (Acosta-Gallegos et al., 1995; Sánchez-Valdez et al., 2004). Both lines are believed to resist drought in different ways (Blair et al., 2009; Beebe et al., 2013; Jimenez-Galindo and Acosta-Gallegos, 2013), and therefore were selected to observe if some SNPs could explain the differences in stress tolerance possibly associated with production-related phenotypes. We used KASP technology to genotype 282 individuals from this population, which were also quantified for phenotypes to perform a SNP-association study. This was followed by functional annotation of significantly associated SNPs showing some possible evidence of which genes could be involved in the drought tolerance.

## Materials and methods

### Plant materials and experimental site

We generated a population of 282 F_3:5_ families derived from the cross between two common bean (*Phaseolus vulgaris* L.) cultivars: Pinto Villa and Pinto Saltillo was used. Both parental genotypes belong to Mesoamerican race Durango (Acosta-Gallegos et al., 1995; Sánchez-Valdez et al., 2004). Population generation was developed from the parental lines to individual F_2_ plants that were bulk advanced to produce F_3_ seed. In the F_3_ generation, 289 individual plants were randomly selfed to develop F_3:4_ families under normal conditions. Then, the 289 families were advanced to F_3:5_, each family was randomly divided into two replicates (two stressed and two watered) and grown in 17 × 17 lattices. For each replicate, the genotypes were sown in 4 m lines with 60 seeds. Two replicates were watered four times (pre-planting, vegetative stage, during the flowering, and pod filling stages) and two replicates omitted the fourth watering, starting the drought stress. All plants were grown in soil with a well-drained sandy loam (Calceric-Cambisol) with slightly alkaline pH (between 7.8 and 8.1) and 0.046% organic matter content.

The experiment was conducted between spring-summer 2010 and 2011 (FR-2010, FR-2011) and autumm-winter 2010 (FT-2010) at the Bajio Experimental Station of National Institute for Forestry, Agriculture, and Livestock (INIFAP), in Celaya, Guanajuato, Mexico (20° 34′ 53″ N, 100° 49′ 16″ W and 1775 m elevation over sea level). The average annual temperatures were 17.7 and 20.8°C and the precipitation were 12, 223, and 30 mm in FR-2010, FT-2010, and FR-2011, respectively (INIFAP Agroclimatic Station Network, http://www.inifap.gob.mx). The stress induced is considered as “terminal” and affected the late stage in development just after flowering time, which directly impacts seed production. At the tropical lowlands on the Pacific, Gulf of Mexico, Central America and the Caribbean, Mesoamerican beans are grown under residual moisture with planting toward the end of the rainy season with soil moisture decreasing toward the end of the crop cycle. Whereas, at the Mexican Plateau the drought is of the intermittent type that often occurs during the reproductive stage, a critical one, and thus a terminal drought treatment mimics up to certain extent drought during the reproductive stage.

We determined relative soil water content (RSWC) using gravimetric methods in two depths: 0–30 and 30–60 cm (Earl, 2003). RSWC average values were 63 and 68% in controls and 25 and 28% in stress. Normal conditions for each replicate were used in both treatments, including mechanical sowing, manual grass removal, and insecticide by aspersion twice (to control the white fly *Trialuroides vaporariorum* Westwood).

Tissue samples were collected after FR-2011. For each genotype, 10 young leaves from different healthy and undamaged plants were collected and frozen in liquid nitrogen for bulked DNA extraction.

### SNP genotyping

After samples were freeze-dried and ground, 100 mg were sent for analysis to LGC Genomics (http://www.lgcgenomics.com, Trident Industrial Estate Pindar Road, Hoddesdon Herts, EN11 0WZ, UK). LGC Genomics previously genotyped the parental lines Pinto Villa and Pinto Saltillo with an initial 1497 SNPs derived from the common variations observed in *P. vulgaris* as part of the international collaboration between INIFAP and the GCP (Generation Challenge Programme, http://www.generationcp.org). They found that 180 SNPs were polymorphic in our parental lines and designed 169 DNA probes of 31 nucleotides long for validation with competitive allele-specific PCR (currently called KASP™) incorporating a FRET quencher cassette (with fluorescence of either VIC or FAM fluorophores) from KBioscience (later renamed LGC Genomics). The 169 probes were mapped to the reference genome and coordinates are reported in Supplementary Table 1. All genotypes found in our population and both parental lines are reported in Supplementary Table 2.

### Phenotypic traits

Days to flowering (DF) and days to physiological maturity (DPM) were recorded as previously described (Acosta-Díaz et al., 2004). DF is the days from seeding to 50% of the plants shows open flowers. DPM is the days from seeding to 90% of the pods changed its green color in alt least 50% of the plants. The reproductive period (RP) was determined by the difference between the days to physiological maturity and the days to flowering (RP = DPM – DF).

Adult plants were collected from a meter row section in each plot and dried at 70°C for 48 h. The weight was recorded to determine vegetative (seedless) plant biomass (PB) (Acosta-Díaz et al., 2004). Similarly, seeds were dried and weighted to obtain seed biomass (SB). The total biomass (TB) is expressed as TB = PB + SB (Acosta-Díaz et al., 2004). All biomass measurements are in g m^−2^. Seed yield (SY) was determined from the total seed weight per area, and recorded in g m^−2^. The weight of 100 seeds (100SW) was obtained from a random sample per experimental unit and recorded in grams. The harvest index (HI) was calculated with the formula HI = SY / TB (Rosales-Serna et al., 2004). The reuse index was calculated as RI = SB / TB. All data recorded is reported in Supplementary Table 3.

### Genome sequences

Genome reference and gene annotation for *P. vulgaris* L. v1.0 was obtained from the Phytozome website (http://www.phytozome.net/). Genome size is 521.1 Mb represented in 708 scaffolds with 27,197 total loci with 31,638 coding-gene transcripts and 4441 alternative splicing transcripts (Schmutz et al., 2014).

### Statistical analyses

Using the probe sequence, each SNP was mapped on to the reference genome with the FASTA aligner (Pearson and Lipman, 1988) obtaining the genomic coordinates for 163 SNPs. This information was analyzed with the R package SNPassoc (González et al., 2007). SNPassoc includes descriptive statistics and exploratory analysis, it also considers in the computation the presence of missing values removing a SNP from the analyses if the observed genotype is below 80%. We used SNPassoc in the calculation of Hardy-Weinberg equilibrium, analysis of association based on generalized linear models (either for quantitative or binary traits), and analysis of multiple SNPs (haplotype and epistasis analysis). In particular, we used the function WGassociation to evaluate each variable for association using a lineal regression model expressed as *variable ~ year + treatment + replicate*. This model allowed us to block the variation effects between cycles and replicates, in particular, the differences in stress induced because we can expect variation given variation in precipitation per year.

We selected all significant SNPs associated with *P* < 0.0003 after Bonferrioni correction testing for variables with codominant, dominant, recessive, overdominant, or log-additive effects.

### Functional analyses

We evaluated how a SNP is associated with a phenotype by functional annotation. We correlated the genomic coordinates with the gene annotation for the *Phaseolus vulgaris* L. v1.0. If the SNP was localized inside a coding exon, we evaluated if this change could generated a negative effect such as a premature stop-codon or a non-synonymous mutation. All genes were functionally annotated by comparison with NCBI-NR database and InterproScan with Blast2GO program (Conesa et al., 2005).

SNP impact on protein product was evaluated using PredictSNP (Bendl et al., 2014), a webservice that integrates several commonly used SNP impact programs and databases such as MAPP, PolyPhen, SIFT, SNAP, PhD-SNP, nsSNPanalyzer, and PANTHER.

## Results

### Experimental data generated

Our experimental design produced two contrasted groups, the watered control-plants, and the terminal-drought plants. Even when rain precipitation was variable per cycle, water was not enough (the minimal water requirement is 400 mm) and the terminal-stress produced a reduction in production as expected (Supplementary Table 3, Supplementary Figure 1). We noted differences in the response in both parental lines, this confirms that the parental lines have differences in the way the stress is tolerated as we are validating at the molecular level (*in preparation*). The genetic recombination in our population allows less differences between stressed and control plants.

### SNP association analyses

The segregation pattern of the 169 polymorphic SNPs was analyzed in the 282 offspring. One hundred sixty three of 169 of these SNPs could be mapped to the reference genome (Supplementary Table 1). A specific position was not found for SNPs: 20, 131, 143, 161, 162, and 169 (Supplementary Table 1), therefore were excluded from the analyzes. The association of each SNP to each one of the quantified variables (SY, 100SW, DF, DPM, RP, PB, SB, TB, RI, and HI) was evaluated with the R package SNPassoc. Supplementary Table 4 shows the *P*-values computed for each possible association between a SNP and the variable in different genetic models tested. With a *P*-value threshold of 0.0003 after Bonferroni correction, 257 statistically significant associations, involving 83 SNPs were identified (Table 1). Briefly, 58 SNPs were associated with flowering time, 44 with seed biomass, 19 with 100-seed weight, 42 with reproductive period, 37 with reuse index, 40 with total biomass, 8 with physiological maturity time, and 1 with harvest index (Table 2).

**Table 1:** **Total SNPs associated to phenotypes by genetic model testing**.

**Trait**	**Codominant**	**Dominant**	**Recessive**	**Overdominant**	**log-additive**	**Total**
SY						
100SW	2	9		8		19
DF	41	4	12	1		58
DPM	2	3	2	1		8
RP	32	1	9			42
PB	6		2			8
SB	37	1	4	2		44
TB	35		4	1		40
RI	35		1	1		37
HI				1		1
Total	190	18	34	15	0	257

Row labels are: SY, seed yield; 100SW, weight of 100 seeds; DF, days to flowering; DPM, days to physiological maturity; RP, reproductive period; PB, plant biomass; SB, seed biomass; TB, total biomass; RI, reuse index; HI, harvest index. Only statistical significant (P < 0.0003) SNPs are counted.

**Table 2:** **SNPs associated to phenotypes**.

**SNP**	**Traits**										**Total**
3		100SW									1
4		100SW									1
5			DF		RP		SB	TB	RI		5
6			DF		RP		SB	TB			4
7			DF		RP		SB	TB	RI		5
9			DF	DPM							2
14						PB	SB	TB	RI		4
16			DF								1
17			DF		RP						2
18						PB	SB	TB	RI		4
19			DF				SB				2
21							SB				1
24			DF		RP		SB	TB	RI		5
25						PB	SB	TB	RI		4
26			DF		RP		SB	TB	RI		5
27			DF		RP		SB	TB	RI		5
28			DF		RP		SB	TB	RI		5
29			DF		RP		SB	TB	RI		5
30			DF		RP		SB	TB	RI		5
31			DF		RP		SB	TB	RI		5
32			DF		RP		SB	TB	RI		5
33			DF								1
34			DF				SB			HI	3
35			DF								1
36			DF		RP		SB	TB	RI		5
38			DF								1
39			DF								1
40			DF								1
41			DF								1
43			DF								1
49						PB	SB	TB	RI		4
54			DF								1
55		100SW									1
56			DF		RP		SB	TB	RI		5
57			DF		RP		SB	TB	RI		5
59			DF		RP		SB	TB	RI		5
60			DF		RP		SB	TB	RI		5
61			DF		RP		SB	TB	RI		5
62			DF		RP		SB	TB	RI		5
63			DF		RP		SB	TB	RI		5
69							SB		RI		2
70		100SW	DF	DPM							3
74			DF		RP		SB	TB	RI		5
75			DF		RP		SB	TB	RI		5
76						PB	SB	TB	RI		4
77			DF		RP		SB	TB	RI		5
79			DF		RP		SB	TB	RI		5
80			DF		RP		SB	TB	RI		5
81			DF		RP		SB	TB	RI		5
85		100SW	DF		RP						3
87		100SW									1
89		100SW		DPM			SB	TB			4
90			DF		RP		SB	TB	RI		5
91			DF		RP		SB	TB	RI		5
94		100SW		DPM							2
97						PB	SB	TB	RI		4
98			DF		RP		SB	TB	RI		5
99		100SW									1
100		100SW									1
104			DF		RP		SB				3
106			DF		RP						2
107			DF		RP						2
109		100SW									1
110			DF		RP						2
112		100SW	DF								2
113		100SW									1
114		100SW	DF								2
115			DF		RP		SB	TB	RI		5
118				DPM							1
120			DF		RP		SB	TB	RI		5
122			DF		RP						2
123			DF		RP						2
128			DF		RP						2
133		100SW		DPM							2
136						PB	SB	TB			3
142		100SW		DPM							2
145			DF		RP		SB	TB	RI		5
148		100SW	DF		RP						3
150			DF		RP						2
155			DF	DPM							2
160						PB		TB			2
165		100SW									1
167		100SW									1
Total	0	19	58	8	42	8	44	40	37	1	257

SNPs with significant association per trail (P < 0.0003) are showed. Trait labels are: 100SW, weight of 100 seeds; DF, days to flowering; DPM, days to physiological maturity; HI, harvest index; PB, plant biomass; RI, reuse index; RP, reproductive period; SB, seed biomass; SY, seed yield; TB, total biomass.

From those, 21 SNPs were observed to be uniquely associated to one variable:

- **100SW**: SNP3 (*p* = 3.42E-05); SNP4 (*p* = 7.69E-05); SNP55 (*p* = 5.01E-06); SNP87 (*p* = 7.23E-05); SNP99 (*p* = 2.80E-06); SNP100 (*p* = 5.86E-05); SNP109 (*p* = 1.04E-04); SNP113 (*p* = 2.26E-04); SNP165 (*p* = 7.23E-04); SNP167 (*p* = 9.10E-04).
- **DF**: SNP16 (*p* = 8.52E-05); SNP33 (*p* = 9.49E-05); SNP35 (*p* = 2.26E-06); SNP38 (*p* = 9.02E-05); SNP39 (*p* = 1.17E-04); SNP40 (*p* = 3.37E-06); SNP41 (*p* = 2.43E-06); SNP43 (*p* = 5.72E-04); SNP54 (*p* = 6.53E-04).
- **DPM**: SNP118 (*p* = 6.09E-04).
- **SB**: SNP21 (*p* = 1.20E-04).

Eighteen SNPs were associated to two variables:

- SNP9, SNP155 with DF and DPM.
- SNP94, SNP133, SNP142 with 100SW and DPM.
- SNP112, SNP114 with 100SW and DF.
- SNP160 with PB and TB.
- SNP17, SNP107, SNP110, SNP122, SNP123, SNP128, SNP150 with RP and DF.
- SNP69 with RI and SB.
- SNP19 with DF and SB.
- SNP106 with DF and RP.

Particularly, high correlations were observed between five variables (DF, PR, SB, TB, and RI); SB, TB, and RI with 30 SNPs (SNP5, 7, 24, 26, 27, 28, 29, 30, 31, 32, 36, 56, 57, 59, 60, 61, 62, 63, 74, 75, 77, 79, 80, 81, 90, 91, 98, 115, 120, and 145).

### SNP functional annotation

SNP probes were aligned in the reference genome and annotated using Blast, InterproScan and Gene Ontology with Blast2GO (Conesa et al., 2005) to identify the gene being impacted by the SNP. Thirty-seven out of 83 SNPs could be identified and associated with a phenotype in a Gene Ontology class (biological process, cellular component or molecular function; BP, CC, and MF, respectively); a detailed table is shown in Supplementary Table 5.

The functional annotation could reveal a putative role of some genes. For example, SNP18, which was strongly associated with biomass, is localized in the gene Phvul.009G226700.1. This gene encodes a putative aldehyde dehydrogenase (NAD), which has been involved in oxidation-reduction process in the response to abiotic stresses (salinity and desiccation) in an abscisic acid (ABA)-mediated response.

Another group of SNPs were associated with kinase proteins, such as SNP9, SNP61, SNP106, and SNP115.

Some SNPs were involved in starch biosynthesis, including genes Phvul.001G230000.1 and Phvul.011G089900.1, containing SNP28 and SNP128, respectively.

We also found several SNPs (14, 38, 40, 76, 90, 97, 99, 100, 104, 107, and 118) that are localized in genes with a putative molecular function of protein binding. Many of them could be associated with signal transduction and may be transcription factors modulating gene expression.

The remaining SNPs had unclear stress-related biological function. For example, SNP16 was localized in actin nucleation; SNP35 was related to postreplication protein repair; SNP49 was in a GTPase; SNP56 with gene regulation in peroxisomes; SNP60 was a putative acyl-transferase; SNP62 and SNP113 were membrane proteins; SNP75, SNP91, SNP120, SNP122, and SNP124 were intracellular transport proteins; SNP80 and SNP98 were associated in transcription regulation; SNP112 was identified as a methyl-transferase in rRNA.

We tested the SNPs functional impact in their respective protein. Seven out of 37 SNPs caused a non-synonymous mutation, but their impact in protein product was neutral or inconclusive as predicted by PredictSNP. Five SNPs are synonymous (silent mutations). The rest, 25 SNPs were localized outside exons making their interpretation harder (Supplementary Table 3).

## Discussion

All plants can be affected by drought at some time during growth because of rainfall variability, water retention in soil, salinity, and extreme temperatures (Chaves et al., 2003). Effects of drought may increase as global climate increases arid areas. Plants have developed response mechanisms to drought stress at the morphological, anatomical, and cellular levels (Chaves et al., 2003; Shinozaki and Yamaguchi-Shinozaki, 2007). Those mechanisms involve increase in water absorption and environment adaptation to reduce leave area and increase root area (Potters et al., 2007; Shao et al., 2008). The responses regulate the expression of several genes including enzymes for antioxidants and osmolite metabolism, signaling proteins such as kinases and transcription factors (Xiong et al., 2002; Chaves et al., 2003). Another physiological resistance mechanism is stoma closure (Taiz and Zeiger, 2006), which is mediate by the phytohormone ABA (Zhang et al., 2006). The ABA is a plant hormone that modulates several stress responses. ABA production in drought is important to initialize early responses to survive the hydric stress (Zhang et al., 2006). As a hormone, ABA is used to communicate with other plants the general stress mode. Besides ABA-related modulation, this gene could be associated with general amino acids production for arginine, histidine, tryptophan, alanine, and proline. Proline is a known amino acid used for osmotic protection for cells in drought stress (Camacho Barrón and González de Mejía, 1998; Ashraf and Foolad, 2007).

Our approach was limited by the size of the population and amount of SNPs tested, making non-optimal for a structured QTL nor as extensive in a tolerant-sensible lines in GWAS. However, the SMA revealed some interesting genes for further validation. In our analysis we found 83 SNPs to be statistically associated to phenotypic traits. After the functional annotation of the respective genes containing those SNPs, we can assign a function to 37 of those genes. Some had a clear function in stress response or tolerance. Plant responses to drought are modulated by diverse physiological mechanisms, which are in general controlled by multiple genes (Foolad, 2004).

The SNP18 is localized in the gene Phvul.009G226700.1, a gene involved in ABA regulation based on its similarity (78%) with ALDH7B4 (AT1G54100) gene in *Arabidopsis* (Kirch et al., 2005). ABA regulates two basic processes in plant development: (1) seed maturation and germination modulated by nutrient accumulation and seed dormancy (Chen et al., 2015); (2) plant responses to abiotic stresses, including drought (Zhang et al., 2006). ABA is a key molecule to modulate and resist drought stress, and its level is balanced with high precision with photosynthesis. In *Arabidopsis*, AtBG1 protein modulates ABA endogenous levels (Hirayama and Shinozaki, 2007); this gene and several *cyp707a* genes are induced by drought and recovery (Saito et al., 2004). Razem et al. (2006) reported putative ABA receptors interacting with FCA protein to modulate flowering time. Another studies in gene promoters and ABA-mutants in *Arabidopsis* showed the role for ABA modulating the drought stress responses (Shinozaki and Yamaguchi-Shinozaki, 2000; Xiong et al., 2002). Besides ABA regulation, this gene is also involved in amino acids biosynthesis. As mentioned before, some amino acids such proline are considered osmolites, therefore their biosynthesis is also increased as a drought response (Ashraf and Foolad, 2007). Proline accumulation, besides osmotic regulation, protects cell membrane and membrane proteins from dehydration and free radicals (Yamaguchi-Shinozaki and Shinozaki, 2006; Ashraf and Foolad, 2007).

We also found SNP28 and SNP128 localized in genes Phvul.001G230000.1 and Phvul.011G089900.1 respectively; these genes are involved in osmotic level regulation by starch biosynthesis. The increase in starch production has been correlated to positive resistance to drought in plants (Krasensky and Jonak, 2012).

In osmotic regulation, water is introduced into the cell by biosynthesis of low molecular weight osmolites (such as carbohydrates, methylamines, free amino acids, and amino acid derivate) and ion accumulation (such as K^+^ and Na^+^) (Cushman, 2001). Osmolites accumulation protects cells from reactive oxygen species (ROS) (Pinhero et al., 1997). Osmolite biosynthesis gene overexpression has been used to protect plant from osmotic stress in several species (Garg et al., 2002; Abebe et al., 2003; Hmida-Sayari et al., 2005; Waditee et al., 2005; Ashraf and Foolad, 2007).

We also identified SNP39, SNP136, and SNP142 that localized in genes Phvul.002G322400.1, Phvul.009G215600.1, and Phvul.010G038200.1, respectively. Those genes are related to ROS, which are generated by an increase in O_2_ photoreduction in chloroplasts (Laloi et al., 2004).

Other commonly induced genes in drought stress are kinases, and we identified SNPs in kinase genes (including SNP9, SNP38, SNP61, SNP99, SNP100, SNP106, SNP115, and SNP142). Kinases are important proteins in signal transport as key proteins for regulation and propagation of stress signals including drought (Umezawa et al., 2006). SNP61 (in the sequence of Phvul.006G097800.1) is associated with myosin heavy chain phosphorylation, which is important in lateral root formation, a common physiological response in this stress. Previous studies in *Arabidopsis* shown a connection between PP2C and a kinase related to SNF1 (sucrose non-fermenting1-related protein kinase or SnRKs), in particular with kinases SnRK2 and SnRK3. Genetic and biochemical studies have demonstrated that a function for SnRK2 is stoma movement, gene expression as response to ABA and ABA responses in drought stress and germination (Fujii et al., 2007). Other kinase identified in wheat is TaSnRK2.8, an SnRK2 (sucrose non-fermenting1-related protein kinase 2) member of wheat. TaSnRK2.8 confers enhanced multi-stress tolerances in carbohydrate metabolism. SNPs in the TaSnRK2.8-A-C region demonstrated that the nucleotide diversity in SNPs was significantly associated with seedling biomass under normal conditions, plant height, flag leaf width, and water-soluble carbohydrate content under drought conditions. Based on the SNP identified, a functional marker of TaSnRK2.8-A-C was developed, that could be utilized in wheat breeding programs aimed at improving seedling biomass and water-soluble carbohydrates, and consequently to enhance stress resistance in wheat (Zhang et al., 2013).

We also found SNP120 in a Late Embryogenesis Abundant Proteins (LEA) gene (Phvul.004G005200.1). LEA genes protect membrane proteins and cell membrane from dehydration; also they are related to osmolite biosynthesis and have antioxidative function (Xiong et al., 2002; Kotchoni and Bartles, 2003).

Another interesting gene is SNP80, localized in the gene Phvul.006G087000.1, a putative transcription factor (TF). The role of TFs is to modulate gene expression binding to specific DNA sites in promoter regions. Previous reports have identified several TFs involved in drought responses (Finkelstein and Lynch, 2000; Nicolas et al., 2014) and their overexpression could increase tolerance to the stress (Garg et al., 2002; Abebe et al., 2003; Shinozaki et al., 2003; Hmida-Sayari et al., 2005; Waditee et al., 2005; Yamaguchi-Shinozaki and Shinozaki, 2005; Ashraf and Foolad, 2007).

Thirty-seven out of 83 SNPs were localized inside coding genes, but functional impact of SNPs is a challenge in each case. We didn't observe a clear functional impact because some SNPs are outside exon or produce synonymous or neutral mutations. It cannot be fully explained without further research.

In the case of common bean (*Phaseolus vulgaris* L.) there are few studies related to genotyping and association to abiotic stresses. Some of these SNPs reported here may be used as markers in MAS to support breeders in the selection of plants with drought resistance without reduction of production, following validation of drought tolerance association in an independent study.

MAS in plants can be implemented quickly and efficiently (Rafalski, 2002). The use of GWAS to identify genes leading to drought tolerance will not identify useful sequence changes if they work via post-transcriptional modifications, which have been shown to have an important role in stress adaptation as in cold (Chinnusamy et al., 2007).

We are working in the development of techniques for easy, quick, and reproducible allele specific marker detection (ASM) to be used in MAS programs for common bean (Collard and Mackill, 2008). Simple techniques such as PCR allele specific amplification can be used in large-scale genotyping of drought-resistant lines as in previous works (Wei et al., 2006; Liu et al., 2012). The efficiency of MAS is reduced by marker-gene recombination, low polymorphism level in parental lines, QTL resolution and genotype by environment interactions. These factors are important to researchers to identify all genetic variants in a population and choose the best markers for MAS (Elshire et al., 2011). Our results can be used as a starting point to develop new marker identification in parental lines in targeted introgression programs.

All genes identified are being studied in our group to understand how their function may provide tolerance in drought and test them in the populations and susceptible lines in future studies.

## Author contributions

EV, MG and PG conceived and designed the project, JA support with expertise and cultivars for common bean lines, EV conducted all experiments, EV and JC performed the data analyzes and biological interpretation. All authors wrote the manuscript.

### Conflict of interest statement

The authors declare that the research was conducted in the absence of any commercial or financial relationships that could be construed as a potential conflict of interest.
