# Supplementary figures and images for: Identification of novel drought-tolerant-associated SNPs in common bean (Phaseolus vulgaris)
Source: Front Plant Sci. 2015 Jul 21;6:546. doi: 10.3389/fpls.2015.00546 (PMC4508514; doi:10.3389/fpls.2015.00546)

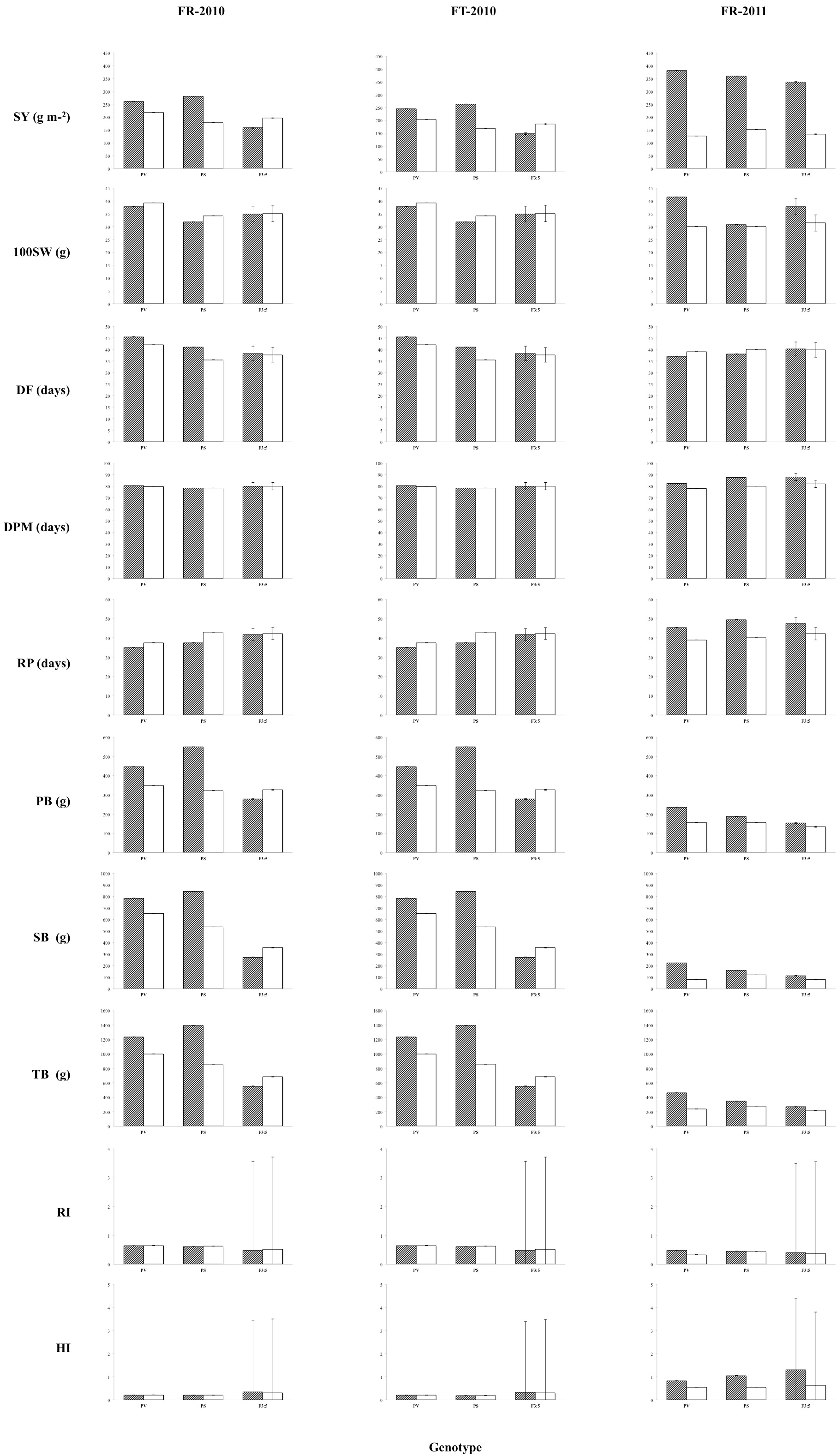

Supplement: Supplementary file 7 [file Image1.TIF]
